# *Helicobacter pylori* and cagA gene detected by polymerase chain reaction in gastric biopsies: correlation with histological findings, proliferation and apoptosis

**DOI:** 10.1590/S1516-31802005000300005

**Published:** 2005-05-02

**Authors:** Katia Ramos Moreira Leite, Elaine Darini, Flavio Canelas Canavez, Claudia Muraro de Carvalho, Cristina Aparecida Troquez da Silveira Mitteldorf, Luiz Heraldo Camara-Lopes

**Keywords:** *Helicobacter pylori*, Pathogenicity island, Polymerase chain reaction, Immunohistochemistry, Apoptosis, *Helicobacter pylori*, Ilhas genômicas, Reação em cadeia da polimerase, Imunohistoquímica, Apoptose

## Abstract

**CONTEXT AND OBJECTIVE:**

The virulence of *Helicobacter pylori* (HP) in gastroduodenal disease is related to pathogenicity islands (*cag*PAI) present in some strains. Infection with *cag*PAI induces IL-8 secretion, increases epithelial cell proliferation and may be important in carcinogenesis. Our objective was to detect HP and the cagA gene (*cag*PAI marker) by polymerase chain reaction (PCR) and to correlate these results to histological findings, epithelial cell proliferation and apoptosis.

**DESIGN AND SETTING:**

Retrospective, at the Surgical and Molecular Pathology Laboratory, Hospital Sírio-Libanês.

**METHODS:**

DNA samples isolated from 164 gastric biopsies were used for HP detection by PCR. *cag*PAI+ was identified in HP+ cases by cagA gene amplification. All cases were submitted to immunohistochemistry to evaluate cell proliferation, and TUNEL to detect apoptosis. Statistical analysis was performed to compare results.

**RESULTS:**

HP was detected in 67.7% of the patients, with good correlation between HP infection and moderate to severe gastritis, gastric ulcer and MALT lymphoma. There was a correlation between *cag*PAI+ strains and severe gastric diseases including cancer. The risk of gastric ulcer, adenocarcinoma and MALT lymphoma was 8.8 times higher for *cag*PAI+ patients. *cag*PAI+ infection was related to higher proliferation rates. The proliferation/apoptosis index was significantly higher for *cag*PAI+ patients.

**CONCLUSION:**

Cell growth deregulation in *cag*PAI+ patients could be demonstrated by the difference in the proliferation index. We believe that this explains the carcinogenic role of *Helicobacter pylori*.

## RESUMO

**CONTEXTO E OBJETIVO:**

A virulência de *Helicobacter pylori* em doenças gastroduodenais está relacionada à presença de ilha de patogenicidade (cagPAI) que ocorre em algumas cepas. A infecção pelo cagPAI induz a secreção de IL-8, aumenta a proliferação epitelial, podendo ter um papel importante na carcinogênese. Nosso objetivo foi detectar HP e o gene cagA (marcador de cagPAI) pela técnica de PCR (*polymerase chain reaction),* correlacionando com os achados histológicos, de proliferação e apoptose.

**TIPO DE ESTUDO E LOCAL:**

Estudo retrospectivo, no Laboratório de Patologia Cirúrgica e Molecular do Hospital Sírio Libanês.

**MÉTODOS:**

DNA isolado de 164 biópsias gástricas foi submetido a PCR para detecção de HP. Os casos positivos foram submetidos a nova reação para identificação do gene cagA. Pela técnica de imunohisto-química foi analisada a proliferação celular e, pela TUNEL, a apoptose.

**RESULTADOS:**

HP foi detectado em 67,7% dos pacientes. Houve correlação entre a presença do HP e o diagnóstico de gastrite moderada ou grave, úlcera e linfoma do tipo MALT. Houve correlação entre *cag*PAI + e a doença gástrica grave, incluindo o câncer. O risco de úlcera, adenocarcinoma ou linfoma MALT para os portadores de cagA+ foi de 8,8. Infecção pelo *cag*PAI correlacionou-se com aumento na taxa de proliferação. O índice proliferação/apoptose foi significantemente maior para os pacientes *cag*PAI+.

**CONCLUSÕES:**

Uma desregulação do crescimento celular nos pacientes *cag*PAI foi demonstrada pela diferença do índice de proliferação, que acreditamos pode explicar o papel carcinogênico da bactéria.

## INTRODUCTION

*Helicobacter pylori* (HP) is a Gramnegative bacterium that colonizes the stomach of approximately two-thirds of the human population and is involved in the pathogenesis of gastroduodenal diseases. In 1994, the World Health Organization and the International Agency for Research on Cancer consensus group classified HP as a type I carcinogen, since its presence is sufficient to induce malignancies without any other co-carcinogens.^1^ The odds ratio for infected people to develop gastric cancer is estimated to be between 1.9 and 3.8, but it may be even higher if infection occurs in young subjects.^2,3^ Recently, Uemura et al. have demonstrated that only people infected with HP (around 3% of them) develop gastric cancer.^4^

The mechanisms involved in carcinogenesis during HP gastric colonization are not fully understood, but the evidence points towards pathogenicity islands (*cag*PAI) as an important virulent factor.^5^ *cag*PAI is a genomic region of approximately 40 Kb, flanked by transposable elements and acquired by horizontal transfer. It contains 31 genes, including six genes named *cag* (cytotoxin associated gene) that are homologues for well-characterized genes encoding type IV export proteins that are specialized in transferring a variety of multimolecular complexes across the bacterial membrane to the extracellular space or into other cells.^6^ These genes are found in the genomes of *Bordetella pertussis*, *Agrobacterium tumefaciens, Escherichia coli, Legionella pneumophila, Rickettsia prowazekii* and *Brucella suis* and show genomic arrangements that are similar to what is observed for *cag* in HP.

Most *cag*PAI protein functions have not been well determined. Nevertheless, their presence is associated with increased interleukin-8 (IL-8) secretion, which is important for chemotaxis and activation of neutrophils.^7^ *cag*PAI is also responsible for (i) remodeling cell surfaces and pedestal formation, (ii) tyrosine phosphorylation of 145-kD host proteins, (iii) activation of the transcriptional factor AP-1, and (iv) expression of the proto-oncogenes c-fos and c-jun by activating the ERK/MAP kinase cascade.^8-10^ Peek et al. have demonstrated that *cag*PAI-positive strains enhance epithelial cell proliferation, increase cell cycle progression from the G1 to G2M phase of the AGS gastric epithelial cell line (ATCC CRL 1739) *in vitro*, and attenuate apoptosis.^11,12^ *In vitro* study has shown that *cag*PAI-positive strains induce gastric damage, while *cag*PAI-negative strains resemble commensal bacteria.^9^

Infections by distinct HP strains vary according to socioeconomic conditions, the patient's age and the country's development status. *cag*PAI-positive strains are prevalent in undeveloped countries and reach nearly 100% in some Asian countries.^13^ In Brazil, Queiroz et al. demonstrated the occurrence of HP *cag*PAI-positive strains in 92% of patients with gastric cancer and in 67.5% of their control group.^14^

We designed a retrospective cross-sectional study to analyze HP infection among patients submitted to gastric endoscopy for dyspeptic symptoms. HP detection and *cag*PAI-positive strain typing were performed by means of the polymerase chain reaction (PCR), using primers for recombinant DNA (rDNA) and cagA (molecular marker for *cag*PAI), respectively. The amplification results were blindly correlated with the histological findings, cell proliferation using Ki-67 (immunohistochemistry) and apoptosis (terminal deoxynucleotidyl transferase nick-end labeling, or TUNEL).

## METHODS

### Patients.

Specimens were collected by gastric endoscopy from 164 patients with dyspeptic symptoms whose ages ranged from 9 to 89 years old (median of 59.5), between January 1998 and July 2001. These patients consisted of 83 females (51%) and 81 males (49%). Random biopsies of global gastric mucosa alterations or fragments of specific lesions were taken and used for histological examination. Specimens were fixed in 10% buffered formalin and embedded in paraffin. Tissue sections of 3 μm in thickness were stained using hematoxylin-eosin (HE) and examined by experienced pathologists (KRML, LHCLandCATSM).

### DNA extraction and PCR.

For each tissue sample, we cut three 10-μm sections from the same paraffin block submitted to HE and transferred them to clean 1.5-ml microtubes. We used disposable microtome blades and cleaned all instruments with xylene after each sample, to avoid cross-contamination. Adding 1 ml of xylene at 80° C and incubating for 30 minutes achieved paraffin removal. The samples were centrifuged at 18,000 g at room temperature for five minutes, and the supernatant was discarded. The pellet was washed twice using 1 ml of xylene at 80° C and twice using 500 μl of 99% ethanol. After the last centrifugation, the pellet was resuspended in 500 μl of digestion buffer (tris-sulfate and ethylenediamine tetraacetic acid, EDTA), added to 10 μl of proteinase K (200 μg/ml, final concentration) and incubated for 12-18 hours at 37° C. The enzyme was inactivated by heat and the proteins were removed by two sequential extractions, using one volume of phenol/chloroform/isoamyl alcohol (25:24:1). The aqueous phase was transferred to a new tube after each extraction. DNA was precipitated from the second aqueous phase by adding 2.5 volumes of ethanol plus 0.1 volume of sodium acetate (3M; pH 5.2) and incubating for 30 minutes at -20° C. The sample was centrifuged at 18,000 g and 4° C for 10 minutes, and the resulting pellet was washed with at least 1 ml of 70% ethanol. The pellet was air-dried for 20 minutes and resuspended in 50 μl of deionized water.^15^ The integrity of each DNA sample was analyzed by amplifying a fragment of -150 bp from the human ß*-actin* gene.

DNA samples were used for HP detection by PCR, using specific primers [5'- CTG GAG A(A/G)A CTA AG(C/T) CCT CC -3' and 5'- GAG GAA TAC TCA TTG CGA AGG CGA -3'] for the HP 16S-rDNA.^16^ The reactions were carried out in 50 μl containing 1X PCR buffer, 0.2 mM of each dNTP, 1.5 mM MgCl_2_, 0.2 μΜ of each primer, 1.25U of *Taq* DNA polymerase and 5.0 μl of DNA sample. The PCR cycle was 95° C for 2 min; (95° C 1 min; 58° C 1 min; 72° C 1 min) 40X; 72° C 5 min; 4° C. If positive for HP, an additional PCR reaction was performed using primers [5'- TCA GAA ATT TGG GGA (A/C)TC AG -3' and 5'- TCA TCA A(A/G)G GA(A/G) TAG GGG TTG -3'] for cagA.^17^ The reactions were carried out in 25 μl containing 1X PCR buffer, 0.2 mM of each dNTP, 3.5 mM MgCl_2_, 0.2 μΜ of each primer, 0.25 U of Taq and 5 μl of DNA. The PCR cyclewas 95° C for 5 min; (95° C 30 sec; 58° C 30 sec; 72° C 45 sec) 40X; 72° C 5 min; 4° C. All PCRs were performed in the Perkin Elmer 2400 thermocycler or MJ minicycler. Aliquots of PCR product were submitted to electrophoresis in 2% agarose gel. The DNA was stained using ethidium bromide for 10 minutes and viewed under an ultraviolet (UV) lamp.

### Immunohistochemical analysis.

Sections of 3 μm in thickness from the same paraffin blocks used for PCR were fixed on adhesive-coated slides. Using the heat retrieval process,^18^ the slides were placed in citrate buffer (1 mM; pH 6.0) and heated three times (eight minutes each) in a domestic microwave oven at high power. The slides were incubated at 4° C for 16 hours, with monoclonal antibody Ki-67 (clone MIB1, Immunotech, Marseilles, France) at 1:50 dilution.

Biotinylated antimouse immunoglobulin G was applied at 1:200 dilution and room temperature for 60 minutes. The slides were rinsed with phosphate-buffered saline (PBS) for 30 minutes, incubated with peroxidase-conjugated streptavidin (strept-ABC kit, Dako) at 1:400 dilution in PBS at room temperature for 45 minutes, and then rinsed again with PBS for 30 minutes. The color was developed by incubating the slides in 0.06% diaminobenzidine (DAB) in PBS for 15 minutes, and the slides were then rinsed in tap water, counterstained with Harris hematoxylin, dehydrated, coverslipped, and reviewed under an optical microscope.^19^ At least 500 cells were counted and the percentage of cells with dark brown nuclear staining was considered to be the proliferation index. The location of the proliferative activity (restricted to the neck, or reaching the isthmus and pit of the gland) was also recorded.

### Detection of apoptosis.

The apoptotic index was assessed by means of the TUNEL technique using the TDT-FragEL kit (Oncogene, Cambridge, Massachusetts, United States), as described by Gavrieli et al.^20^ Briefly, 3-μm sections of the same block used for immunohistochemistry and PCR were placed on adhesive-coated slides, deparaffinized in xylene, and rehydrated in alcohol. After cell membranes were made permeable by treating the slides with proteinase K diluted 1:10 in 10 mM Tris, at pH 8.0 and room temperature for 20 minutes, endogenous peroxidase was inactivated by applying 3% H_2_O_2_ at room temperature for five minutes. Labeling was performed by incubating the slides in a humidified chamber at 37° C for 1.5 hours with the terminal deoxynucleotidyl transferase (TdT) reaction mix from the kit, which contains labeled and unlabeled deoxynucleotides as well as the terminal deoxynucleotidyl transferase enzyme. The reaction was stopped by applying 0.5 M ethylenediamine tetraacetic acid (EDTA), at pH 8.0 and 37° C for five minutes, and blocked by applying 4% bovine serum albumin in phosphate-buffered saline at room temperature for 10 minutes. The peroxidase-conjugated streptavidin, diluted 1:50 in blocking buffer, was applied at room temperature in a humidified chamber for 30 minutes. The slides were then incubated with DAB, counterstained with methyl green, and coverslipped. At least 200 cells were counted, and the percentage of stained cells was considered to be the apoptotic index.

### Statistical analysis.

To verify significant relationships between variables, the chi-squared test was used for qualitative variables and the non-parametric Mann-Whitney test was used for quantitative variables. Logistic regression was used for multivariate analyses and to identify the odds for developing severe gastric diseases or malignancies. The p-values were two-sided and were considered significant when less than 0.05. The tests were performed using the Statistical Package for the Social Sciences (SPSS) software, version 10 (SPSS, Inc., Chicago, Illinois, United States).

## RESULTS

### Patients.

HP was detected in 111 patients (67.7%). The infection affected both genders equally: 67.9% of females and 67.5% of males. Regarding the age distribution, HP was detected in 46.2% (33.3% cagA+) of patients from 9 to 30 years, 76.9% (43.0% cagA+) from 31 to 50 years, 75.0% (41.7% cagA+) from 51 to 60 years, 64.9% (45.8% cagA+) from 61 to 70 years and 62.8% (33.3% cagA+) from 71 to89 years.

### HP, cagA and histology.

The histology, HP and cagA (a molecular marker for cagPAI) results are in Table 1. The PCR was positive in 11 cases (34.4%) of normal mucosa or mild gastritis, in 44 specimens (86.3%) of moderate to marked gastritis, in 32 cases (74.4%) of gastric ulcer, in 4 cases (50%) of atrophic gastritis and in 5 cases (100%) of mucosa-associated lymphoid tissue (MALT) lymphomas (p < 0.0001). Out of 111 HP-positive specimens, 45 (40.5%) were cagA-positive, of which 20 (44.4%) were from females and 25 (55.6%) from males. The cagA was positive in three cases (27.3%) of mild gastritis, seven (15.9%) moderate to marked gastritis, 23 (71.9%) gastric ulcer, two (50%) atrophic gastritis, one with and one without intestinal metaplasia, and four (100%) adenocarcinoma (p < 0.0001).

**Table 1:** Polymerase chain reaction results: *Helicobacfer pylori* and cagA (molecular marker for cagPAI) status and diagnosis from gastric biopsiestaken randomicallyfrom 164 patients

Diagnosis	*Helicobacter pylori*			cagA		
	Negative n (%)	Positive n (%)	Total n	Negative n (%)	Positive n (%)	Total n
Normal mucosa or mild gastritis	21 (65.6)	11 (34.4)	32	8 (72.7)	3 (27.3)	**11**
Moderate to marked gastritis	7(13.7)	44 (86.3)	51	37 (84.1)	7 (15.9)	**44**
Gastric ulcer	11 (25.6)	32 (74.4)	43	9 (28.1)	23 (71.9)	**32**
Adenocarcinoma	2 (33.3)	4 (66.7)	6	0	4 (100)	**4**
MALT lymphoma	0	5 (100.0)	5	2 (40.0)	3 (60.0)	**5**
Hyperplastic polyp	8 (42.1)	11 (57.9)	19	8 (72.7)	3 (27.3)	**11**
Atrophic gastritis	4 (50.0)	4 (50.0)	8	2 (50.0)	2 (50.0)	**4**
**Total**	**53 (32.2)**	**111 (67.7)**	**164**	**66 (59.5)**	**45 (40.5)**	**111**
	p < 0.0001			p < 0.0001		

*MALT = mucosa-associafed lymphoid fissue.*

### Proliferation.

**Figure 1:**
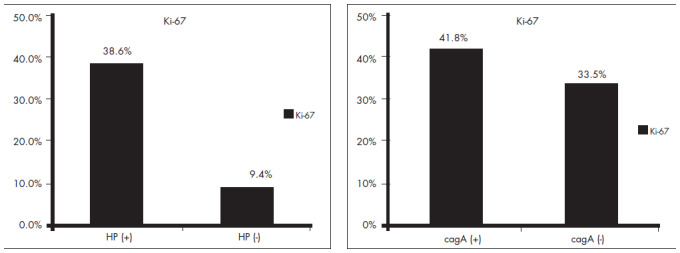
Median of the percentage of epithelial cell proliferation measured by Ki-67, in *Helicobacfer pylori* and cagA (molecular marker for cagPAI) positive and negative cases, in 164 patients with dispeptic symptoms.

There was a significant difference between the HP-positive and HP-negative groups regarding cell proliferation. The mean and median of the proliferation index were 15.3% and 9.4% (range: 1.4 — 49%) for HP-negative cases and 37.5% and 38.6% (range: 1.0 — 80%) for HP-positive specimens (p < 0.0001). The same significance was noted regarding the cagA status. The mean and median of the cell proliferation index were 33.7% and 33.5% (range: 1.0 — 68%) for cagA-negative specimens, and 43.0% and 41.8% (range 5.0 — 80%) for cagA-positive cases (p = 0.015) (Figure 1).

Considering the distribution of proliferating cells, it was evident that in HPpositive cases the proliferative activity was not restricted to the neck of the glands, but extended throughout the isthmus and base. In cagA-positive cases, stained cells reached the pit of the gland and the surface of the epithelium.

The highest proliferation rate was in malignancies, with medians of 42.0% and 41.8% for adenocarcinomas and MALT lymphomas, respectively. The lowest proliferative rate was in the normal epithelium, with a median of 20.3%.

### Apoptosis.

Differing from the proliferative index, apoptosis was not statistically different between the HP-positive and HP-negative groups. The mean and median of apoptosis were 18.7% and 13.8% (range: 1.0 — 58.6%) for HP-positive cases, and 16.4% and 11.3% (range: 0.2 — 64%) for HP-negative cases (p = 0.068). The apoptotic index, although higher in cagA-negative specimens, did not reached statistical significance. The means and medians for cagA-positive and cagA-negative cases were, respectively, 14.7% and 11.9% (range: 1.0 — 58.6%), and 21.3% and 16.3% (range: 2.5 — 58.5%) (p = 0.061).

The distribution of apoptotic bodies was randomized in the epithelium, and was unrelated to HP or cagA status.

Considering the diagnosis, the highest apoptosis rate was in atrophic gastritis (median of 5.2%) and the lowest was in normal gastric mucosa (median of 1.4%).

#### Proliferation-to-apoptosis ratio.

**Figure 2:**
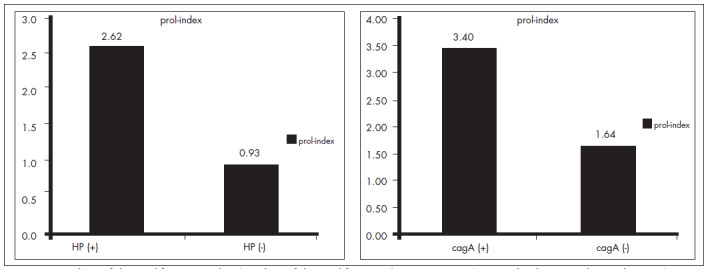
Median of the proliferative index (product of the proliferation/apoptosis ratio), in *Helicobacter pylori* and cagA (molecular marker for cagPAI) positive and negative cases, in 164 patients with dyspeptic symptoms.

The final proliferative index is the prolifera- tion/apoptosis ratio (P/A), which drives the cell growth rate. This ratio was significantly higher in HP-positive specimens, with a mean of 3.63 (median 2.62; range: 0.0222.84), than in HP-negative specimens, with a mean of 2.10 (median 0.93; range: 0.05-11.67) (p < 0.0001). The same results were found for cagA status. CagA-positive cases had higher levels of P/A, with a mean of 4.09 (median 3.4; range: 0.57-13.02), than did cagA-negative specimens, with a mean of 2.33 (median 1.64; range: 0.0222.84) (p = 0.002) (Figure 2).

#### Relationship between cagA status and gastric disease.

The logistic regression analysis showed a risk of developing severe gastric disease, gastric ulcer, adenocarcinoma and MALT lymphoma that was 8.8 times higher for cagA-positive strains. The other variables, including sex, proliferative activity, apoptotic index and P/A ratio were not independent factors for predicting marked gastric damage.

## DISCUSSION

Using PCR we detected HP colonization in 68% of our patients. For the age range from 8 to 30 years, the incidence of infection was 46%. This was similar to what was reported by Oliveira et al., who found HP infection in 34% out of a pediatric population of 241 patients aged one month to 18 years.^21^ We have shown that the incidence of HP detection was 75 to 77% for patients aged 31 to 60 years. This rate is very similar to what has been reported by others in Brazil and other countries in Latin America.^22,23^ In Brazil, Rocha et al. found an HP infection rate of 62% to 66% among 320 adult patients.^24^ In Chile, Hopkins et al. found HP colonization in 62 to 69% of patients, independent of their socioeconomic status.^25^

The increasing infection rates over the course of life suggest that there are no natural mechanisms for bacterial clearance and that the contamination persists. The drop in HP infection in our study after the age of 60 years could be explained by the development of atrophic gastritis. Seventy-five percent of the atrophic gastritis in our population was diagnosed after the age of 61 years. Atrophy changes the gastric environment by decreasing the acid secretion, thus creating unfavorable conditions for bacterial survival.

The strong correlation we observed between the HP infection detected by PCR, and the histological findings from gastric biopsies is noteworthy. PCR was positive in 86% of moderate to marked gastritis cases, 67% of adenocarcinomas and 100% of MALT lymphomas, but only in 34% of normal gastric mucosa and mild gastritis cases. It is already known that only a minority of patients present acute symptoms related toHP infection, but 80% of them will evolve into chronic gastritis if HP infection persists.^26^ The lifetime risk for developing gastric ulcer in a person infected by HP is estimated as 3% in United States and reaches 25% in Japan.^27^ HP infection is also associated with gastric cancer, as shown by Uemura et al. who, when following up patients for almost 8 years, detected adenocarcinoma in 3% of HP-infected individuals.^4^ The correlation between HP infection and dyspeptic symptoms is controversial. Some authors have reported the relief of symptoms and very low rates of HP infection recurrence in a population treated with omeprazole and antibiotics, in comparison with patients treated with H2 inhibitors only.^28^

The consequences of infection depend on a variety of factors relating to the host and the bacterium itself. Our data show that the presence of cagA (molecular marker for *cag*PAI) is important in the development of severe gastric disease, including gastric ulcer (72%), adenocarcinoma (100%) and MALT lymphoma (60%). While histology revealed only mild or severe gastritis, the detection of cagA ranged from 16% to 27%. The multivariate analysis showed that the risk of developing gastric ulcer or malignancies when cagA was present was 8.8 times greater. Our findings are similar to those in the literature that correlate *cag*PAI strains with the severity of gastric disease.^29-31^

*In vitro* and *in vivo* studies have shown that the presence of the genes in *cag*PAI, including cagA, cagE (picB), cagG, cagH, cagI, gagL and cagM, is required for the release of pro-inflammatory cytokines such as IL-8.^32^ In *cag*PAI, there are a number of genes responsible for coding for proteins. These build up a type IV secretion system that is used to introduce proteins directly into the epithelial host cells. CagA is one of these proteins and, when injected into the cell, it is phosphorylated by tyrosine and binds to the src-homologous region 2 domain containing proteins, thereby forming a signaling complex that promotes the reorganization of cortical actin and components of the apical membrane.^33^

In our study, the proliferation index was significantly higher among patients infected by HP, including the cagA-positive strain. Peek et al. have already shown progression of gastric epithelial cells from Gl to G2-M *in vitro*.^l2^ One interesting observation we have made concerns the switch of proliferation location, which was not restricted to the neck of the gland, but extended to the isthmus and surface of the foveola. Our findings corroborate those described by Testino et al., who maintained that the retarding of cell differentiation can collaborate in metaplastic changes and neoplastic development.^34^

Xia and Talley suggested that HP could be involved in the development of gastric cancer by inducing apoptosis of epithelial cells, thus leading to atrophic gastritis and achlorhydria.^35^ As well as the fact that we did not find differences in the apoptotic index in our cases, we showed an increase in apoptosis in HP-positive patients, and a decrease in the cagA-positive strain. Moss et al., studying a smaller number of patients, had similar results: higher proliferation in the gastric epithelium of HP-infected patients and a decrease in apoptotic index in those harboring the cagA-positive HP.^36^

The final proliferation index is the P/A ratio, and we were able to show a significant increase in this index among patients infected by HP and those who were cagA-positive. The median P/A ratio for HP-positive patients was 2.62, while it was only 0.93 for HP-negative cases. For cagA-positive strains, the figures were 3.40 and 1.64 for HP-positive and HP-negative cases, respectively. However, the gastric epithelial cells from patients infected by HP, and especially from those with the cagA-positive strain, probably have an advantage in their growth, which may explain the relationship between HP infection and the development of cancer.

PCR is a powerful tool for the diagnosis of HP infection. It is highly sensitive and capable of detecting very small quantities of bacteria, even when specific treatment is underway. It is also very specific, since the primers chosen have been tested on more than 35 different culturing microorganisms and have only been found to amplify HP, *Helicobacter acinonyx* and *H. nemestrinae* (these last two species have never been found in humans).^16^ An additional advantage is the possibility of characterizing the bacterial genotype, including the presence of *cag*PAI, vacA and bab2, which directly influence the performance of HP.

Several other tests are currently being used for detecting HP infection. Every test presents advantages and disadvantages that hamper the definition of a gold standard method. The tests can be divided into two categories based on their invasiveness: non-endoscopic and endoscopic tests. The former group includes methods such the detection of antibodies in the blood by the enzyme-linked immunosorbent assay (ELISA) and the ^13-14^carbon-urea breath test (CUB).^37^ ELISA methods show sensitivity ranging from 76 to 84% and specificity from 79 to 90%, but cannot distinguish between active and recently treated infection.^38^ This limitation is not observed in the CUB method, which only detects active bacteria. CUB sensitivity, however, is reduced with the use of proton pump inhibitors, antibiotics and compounds containing bismuth, which decrease the quantity of HP or its urease activity.^39^

Examples of invasive methods are the rapid urease test (RUT), histology and HP culturing. The RUT presents good sensitivity and specificity, and it is cost-effective and fast, but it is influenced by previous treatments.^40^ Histology is very sensitivity and allows the identification of concurring mucosa pathology, but it is a subjective method that can produce variable results due to differences in the pathologist's criteria. In this method, better results are achieved if special stains are used. Bacterial culturing allows the determination of an antibiotic sensitivity profile, but needs a long time for definitive results. Moreover, sampling error, recent use of antibiotics and contamination of the specimens with glutaraldehyde during biopsy acquisition^41^ also compromise bacterial culturing.
